# Investigating the Effect of the State, Stability, and Change in Deep Approaches to Learning From Kindergarten to Third Grade: A Multilevel Structural Equation Modeling Indicator-Specific Growth Model Approach

**DOI:** 10.3389/fpsyg.2022.852508

**Published:** 2022-03-08

**Authors:** Chung Chin Wu

**Affiliations:** Department of Early Childhood Education, National Pingtung University, Pingtung, Taiwan

**Keywords:** approaches to learning, kindergarten, lower elementary school, structural equation modeling indicator-specific growth model, longitudinal changes

## Abstract

Adopting deep approaches to learning can have a profound impact on learning outcomes. The extent of change in the learning approach could be attributed to the effect of contextual factors (e.g., instruction). After a substantive review, it was found that research interested in investigating the longitudinal effect of deep approaches to learning on learning outcomes were rarely directly concerned with the longitudinal state and trend of the approach itself. Moreover, the limitations of past analytical methods (e.g., using a single indicator to represent deep approaches to learning at each point in time), has not been appropriately acknowledged. This study examined deep approaches to learning in students from kindergarten to third grade using a multilevel structural equation modeling indicator-specific growth model (MSEM-ISGM). This was used to highlight the methodological issues (e.g., consider four indicators separately at each wave) to investigate the effect of context and the states, stability, and changes in deep approaches to learning over this developmental period. The current study comprised of a large-scale sample of 21,490 kindergarteners in kindergarten. Results showed: (1) there was a contextual effect on the longitudinal changes of deep approaches to learning; (2) deep approaches to learning was high but showed considerable individual differences; (3) most indicators relating to deep approaches to learning declined (however, one increased), whereas the trend were relatively stable over time. Corresponding suggestions were proposed at the end of this article.

## Introduction

Approach to learning is defined as a child’s individual motivation and observable learning strategy revealed while engaging in learning activities. This concept can be categorized as either surface, achieving, or deep approaches (McWayne et al., 2004). Deep approaches to learning was found to have a close relationship with academic achievement (Biggs and Moore, 1993; Goh et al., 2012) and can be identified through a student’s active engagement and intention to understand the meaning of materials, work independently, persevere with difficult tasks, and attentiveness (Entwistle et al., 2000; Razza et al., 2015). Within a review, researchers outlined the encouraging and discouraging factors in stimulating the adoption of deep approaches to learning (Baeten et al., 2010). This review not only highlighted the importance of deep approaches to learning, but implied that effective contextual factors (e.g., instruction) may contribute to cultivating the approaches. Goto et al. (2018) found that participating in educational activity (scientific-related activities) can have a positive effect on the deep approaches to learning, suggesting a context effect on which. However, the extent of the effect of contextual factors was unclear. In addition, most of research relating to deep approaches to learning focused primarily on upper elementary school levels or further academic levels (García et al., 2016; Hu and Yeo, 2020; Zakariya et al., 2020, 2021). The empirical research in earlier educational stages and involving transition from one to the other educational system (i.e., from kindergarten to lower elementary school) remained scarce. Although, Sung and Wickrama (2018) has investigated approaches to learning from kindergarten year to first grade, but the context effect on which was still unable to be identified by their methodological approaches. Consequently, it is important to investigate the extent of the effect of contextual factors on deep approaches to learning involving a transition from kindergarten to lower elementary school levels.

The importance of developing deep approaches to learning early is highlighted through studies in which kindergarteners who adopted deep approaches to learning were found to have a high level of achievement across elementary school (McClelland et al., 2006; Li-Grining et al., 2010; Morgan et al., 2011; Razza et al., 2015). However, a few researches found there was no effect of the deep approaches to learning on academic performance (García et al., 2016; Ardura and Galán, 2019; Zakariya et al., 2021). It may imply that some methodological problems resided in former empirical findings, and it could not be identified and resolved due to limitations of the analysis method itself they used. For example, Li-Grining et al. (2010) averaged the four scales of deep approaches to learning to create a single composite score and used this single indicator in their traditional growth model analysis, and they mentioned they were unable to generate the exact internal consistency of ATL measure without the item-level data. In fact, the deep approaches to learning were composed of both trait-like (i.e., persistence) and state-like components (i.e., attentiveness) which may be, respectively, considered as time-consistent (more independent of context) and time-varying variables (more depend on context). It was more reasonable to separately consider each component in the deep approaches to learning rather than to aggregate them to form a single indicator because they may have different states at different time points/education levels and different trend over time. However, above considerations have not been correctly addressed. In addition, both the extent of individual differences for each component in the deep approaches to learning and the extent of the change and stability of which were also remained unclear. Until now, all above mentioned considerations have not been resolved by using either typical growth model (McClelland et al., 2006; Li-Grining et al., 2010; Morgan et al., 2011; Sung and Wickrama, 2018; Ardura and Galán, 2019) or regression analysis in related studies (Razza et al., 2015; García et al., 2016). Consequently, it was important for study to separately consider each component in the deep approaches to learning in analysis, and further clarified their states, changes, and stabilities. This could enhance future research relating to the deep approaches to learning, and be beneficial for teachers to identify appropriate time point to implement an instructional intervention program to promote it or prevent its decline, and reduce individual differences on this adaptive approach.

By using single indicator to represent approaches to learning in typical growth model, Sung and Wickrama (2018) found that approaches to learning retained relative high level and slightly growth in positive way, and it was showed considerable individual difference and relative stability from kindergarten to first grade. However, this finding was inconsistent to those studies in the learning motivation literature. Specifically, it has been found that pupils become less oriented toward mastery goals (e.g., challenge new things, a high level of effort and persistence) with increased age, as well as less intrinsically motivated throughout elementary and middle school (Anderman and Midgley, 1997; Gottfried et al., 2001; Meece and Miller, 2001; Bouffard et al., 2003). In general, a high mastery goal orientation is maintained from first to third grade (Bong, 2009), but begins to deteriorate from third to fifth grade (Meece and Miller, 2001). It would be interesting to identify whether these findings are similar in regard to deep approaches to learning once the methodological problems are addressed. New analysis method was introduced to achieve following two purposes of this study:

1. To increase understanding of the effect of the context on deep approaches to learning.
2. To examine the states, stability, and trend of such learning from kindergarten to third grade.

## Deep Approaches to Learning

Approaches to learning is a collective construct encompassing both an individual’s motive and their learning strategies, which is reflected in the way students engage in their learning task (Biggs, 1988). There are three main kinds of approaches to learning: surface, achieving, and deep (Biggs et al., 2001). Students adopting deep approaches to learning are actively engaged and interested in studies, intend to understand the meaning of the material, work independently, persevere with difficult tasks, and pay attention well in class, and it is considered the most adaptive to learning outcomes (Entwistle et al., 2000; Razza et al., 2015).

Students with deep approaches to learning are generally eager to understand learning material, intrinsically motivated to engage in activities, and interested in learning new things (Laitinen et al., 2017). In addition, such students attempt to master their work independently, therefore adopting a mastery orientation to their learning and showing engagement, high levels of effort, and a commitment to learn (Sideridis, 2005). The deep approaches to learning is more beneficial to learning than the other two, therefore deserving more discussion in terms of its stability and changes due to its profound impact on learning (Harter, 1981; Blair, 2002).

Most research related to deep approaches to learning have focused on its relationship to achievement and involved mostly students above the age of six (Fryer and Vermunt, 2018; Khan et al., 2018; Rentzios et al., 2019; Chan et al., 2021; McWatt et al., 2021; Piumatti et al., 2021). For example, Duff et al. (2004) examined the cross-sectional relationship between deep approaches to learning and grade-point average among undergraduate students using a structural equation modeling analysis. Teoh et al. (2014) examined nine hypothetical learning approaches (including deep, surface, and achieving approaches) by using person-based cluster analyses. There has also been regression analyses and growth curve analysis regarding kindergarten children, where deep approaches to learning positively predicted reading and numeracy achievement throughout elementary school (McClelland et al., 2006; Li-Grining et al., 2010; Morgan et al., 2011; Razza et al., 2015).

In summary, the characteristics of deep learning approaches comprise a set of motivational beliefs and strategies. This includes an interest in learning challenging or new material, independent work completion, persistence and learning attendance. After a substantive review, it is apparent that limited evidence has been presented in terms of the states, stability, and changes in deep approaches to learning from kindergarten to third grade.

### The State, Stability, and Change in Deep Approaches to Learning

There is a significant gap in the current research in regard to the stability and changes in learning approach within primary school students, with cross-sectional or longitudinal examination narrowly focused in higher education. For example, Zeegers (2001) found that undergraduate students’ deep and surface approach to learning was stable across five waves within a period of two and half years.

The high degree of deep approaches to learning within kindergarteners has been demonstrated through numerous studies. For instance, a series of experiments found that when kindergarteners were faced with constant failure on a certain task, they would persevere with the arduous task and increase efforts (Rholes et al., 1980; Dweck and Elliot, 1983; Harter, 2012). More recently, researchers conducted a person-based analysis with longitudinal data and found that most kindergarteners aged between four and six persisted in completing learning tasks, were motivated to learn new information and had a preference for challenging tasks (Laitinen et al., 2017). In addition, Bong (2009) found that students in first to fourth grade were more concentrated on learning a challenging or new task in comparison to fifth to ninth grade. To extend on this, it was suggested that students’ deep approaches to learning may be higher before third and fourth grade These evidence implies that deep approaches to learning were (1) less susceptible to context and (2) remained high and stable from kindergarten to third grade.

### Methodological Consideration

A few studies introduced slightly different scales to measure kindergarteners’ approach to learning (McClelland et al., 2006; Li-Grining et al., 2010; Morgan et al., 2011; Razza et al., 2015). In these studies, kindergarteners’ approaches to learning was measured either by the Approaches to Learning Scale (Li-Grining et al., 2010; Morgan et al., 2011; Razza et al., 2015) or by Cooper-Farran Behavioral Rating Scales (McClelland et al., 2006). Latent growth modeling (LGM) (McClelland et al., 2006; Li-Grining et al., 2010; Morgan et al., 2011) or multiple regression (Razza et al., 2015) were, respectively, implemented in these studies to analyze data through the use of composite scores (e.g., a sum of all item scores to form a single indicator).

There are three primary concerns within these studies regarding the analysis of deep approaches to learning. Firstly, both LGM and multiple regression analysis use a composite score as a single indicator, ignoring the fact that items are not measured perfectly and each item may contain considerable measurement errors. Composite scores represented as a single indicator does not take into consideration multiple measurement errors within the analysis process (Wang and Wang, 2012). Accordingly, results may be questionable without understanding the effect of measurement errors. In addition, it has been found that the single-indicator in LGM has less statistical power for detecting individual differences in changes over time compared to multiple-indicator approaches (von Oerzen et al., 2010).

Secondly, the single-indicator in LGM implies that the construct under study is perfectly trait-like and is not dependent on situational influences (Geiser et al., 2013). Specifically, former researches used only one indicator to represent approaches to learning in each wave, which resulted in an inability to decompose the variances formed from contextual factors, traits, or measurement errors. In contrast to the composite approach used in previous studies, separately considering each item throughout the analysis process is more suitable for examining whether the context may exert effect on the states and stability of deep approaches to learning.

Thirdly, deep approaches to learning comprises of a motivational and strategic component, which implies that observed variables may have different psychological origins. This means that multiple items should be analyzed simultaneously within the model. Through this analysis, multiple items should reveal similar trends if all measuring the deep approaches to learning construct and if originated from identical psychological origins. In contrast, if items directed toward deep approaches to learning show inconsistent tendencies, this may suggest different psychological origins. The indicator-specific growth model (ISGM), which relaxes the assumption of perfectly unidimensional indicators in the context of latent growth modeling, was recommended for this kind of investigation. The intercept and slope factors in the ISGM are indicator-specific. This means that indicators can differ in scaling, initial trait level, and rate of trait change (Geiser et al., 2013).

Within past research, deep approaches to learning was assumed to be a trait-like concept which is less susceptible to context. To disprove this assumption and analyze the effect of the learning context, a multilevel SEM extension of ISGM (MSEM-ISGM) model should be used as it is capable of measuring situational effect on fixed traits and long-lasting trait changes at level two (Geiser et al., 2013). The MSEM-ISGM is also capable of modeling the effects of measurement error, common latent state residual factors, the time-invariant variance and factor loadings, and time at level one. The overall means and variations of the states and trends of deep approaches to learning were modeled at level two. As a result, the MSEM-ISGM was a better approach to investigate the effect of context on deep approaches to learning, and the states, stabilities and changes of which.

## The Current Study

It can be seen through the current literature that deep approaches to learning has a profound impact on academic outcomes. However, the effect of context on this approach, as well as the states, longitudinal stability and whether changes occur were rarely documented. Through empirical evidence, it is suggested that deep approaches to learning may start to decline after third grade (i.e., Bong, 2009). However, no direct evidence indicating the tendency from kindergarten to third grade. It was assumed that deep approaches to learning are trait-like and remain high and stable from kindergarten to third grade. Moreover, current related longitudinal studies may be questionable because of methodological issues, such as using a composite score as a single indicator in analysis, which may underestimate the effect of the errors and confound the effect of the context.

Based on a large-scale and multi-wave kindergarten sample, the current study aims to investigate the effect of context on deep approaches to learning and examine the states, longitudinal stability, and changes in student’s approaches to learning. Based on a literature review, three hypotheses are as follows: in general, from kindergarten to third grade (1) learning context will exert trivial effect on deep approaches to learning. (2) deep approaches to learning will be high and show considerable individual differences. (3) deep approaches to learning will be stable.

## Methodology

### Participants

The participants of this study were selected from the Early Childhood Longitudinal Study, Kindergarten Class (ECLS-K). ECLS-K is sponsored by the National Center for Education Statistics (NCES) within the Institute of Education Sciences (IES) in the United States Department of Education. This large-scale employed a multistage probability sample design to select a nationally representative sample of children attending kindergarten, and the primary sampling units (PSUs) were geographic areas. 100 census regions were included, there were 18, 25, 34, and 23 located, respectively, in the Northeast, Midwest, South, and West. The second stage units were schools within sampled PSUs, and the third- and final-stage units were children within schools. A total of 1,277 kindergartens were selected, and they were composed of 914 public and 363 private kindergartens. There were 443 kindergartens enrolled less than 50 children, 461 kindergartens enrolled the amount of children between 50 and 99, and 373 kindergartens enrolled more than 100 children. The majority of ethnicity were white (59%). Male and female were 51 percent and 49 percent, respectively. The mean age of kindergarteners was 68.50 (*SD* was 0.032), and the mean of the child’s household size was 4.52 (*SD* was 0.010). 45 percent of children attended half-day kindergarten and 55 percent attended full-day kindergarten programs (National Center for Education Statistics, 2001; Rathbun and West, 2004). This longitudinal survey study followed the same children from kindergarten through to eighth grade and is considered a nationally representative sample with children from both public and private schools attending both full-day and part-day kindergartens. The ECLS-K included public-use data of 21,490 kindergarten to eighth grade students throughout the nation. Children in the ECLS-K were recruited sequentially in the fall and the spring of kindergarten, the fall and spring of first grade, the spring of third grade, the spring of fifth grade, and the spring of eighth grade. The fall of first grade data was discarded because NCES only collected such data from a small (30%) sub-sample of children during this survey wave (Tourangeau et al., 2009). Finally, kindergarteners in the fall (wave 1) and in the spring (wave 2), first grade students in the spring (wave 3) and third grade in students in the spring (wave 4) were included in this analysis.

### Instruments

Deep approaches to learning relates to a child’s learning-related motive and strategy, which was measured using teacher ratings on the Approaches to Learning subscale, a modified version of the Social Skills Rating System (Gresham and Elliot, 1990). NCES refers to the modified version of the SSRS as the Social Rating Scale (SRS). The Approaches to Learning subscale seeks to measure a child’s eagerness to learn, work independence, task persistence and attentiveness. Following four items which focus on deep approaches to learning were adopted in this study: (1) shows eagerness to learn new things. (2) works independently. (3) persists in completing tasks. (4) pays attention well. Teachers rated how frequently each statement was observed on children in their classroom using a 4-point scale: 1 (never), 2 (sometimes), 3 (often), and 4 (very often). Teachers’ observations or general impressions to young children may include children’s behaviors in the learning area as well as in the leaning tasks or activities, and teachers’ assigned task (i.e., assign kindergarteners to make an artifact they taught). It was commonly used approach when young children were included in the study, and it was assumed that children’s “inner” part of the deep approaches to learning may be inferred from observable external behaviors (McDermott et al., 2002; George and Greenfield, 2005; Betts and Rotenberg, 2007; Greer et al., 2015). Scores for each item were not added to form a single indicator in every single wave. In other words, the scores of each four items remained separate to undertake MSEM-ISGM.

### Analysis

The intra-class correlation (ICC) was used to evaluate the dependence of deep approaches to learning for each item across time. Items fulfilled the requirements of ICC when they were greater than 0.05 (Heck, 2001; Dyer et al., 2005), suggesting that the analysis should take into consideration a two-level structure. Following this, a conventional single level growth model (GM) and MSEM-ISGM were compared to decide which is more suitable to investigate the effect of context and states, stability, and changes in deep approaches to learning (Kaplan, 2009).

**FIGURE 1:**
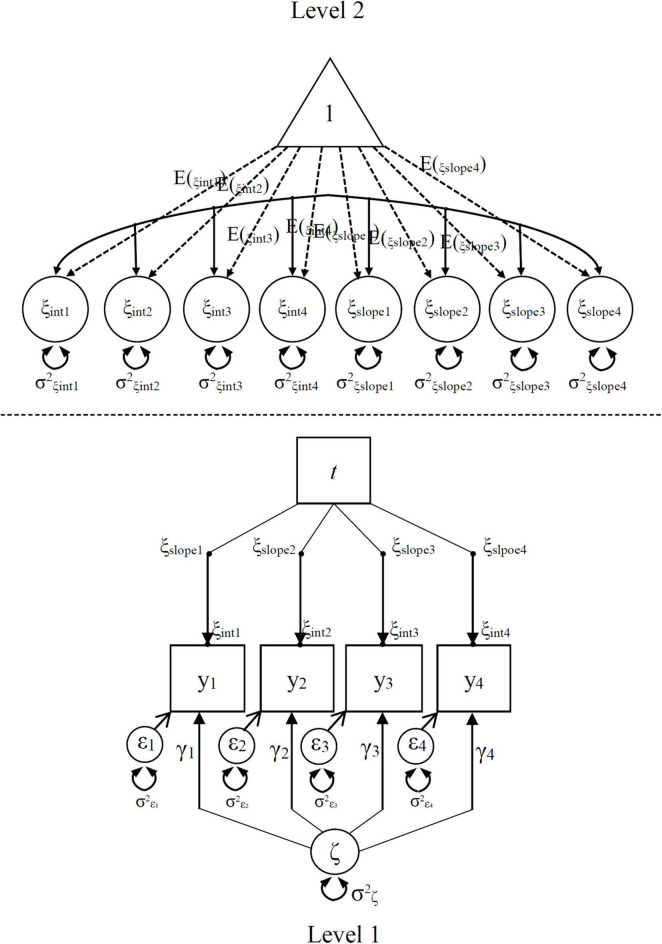
The multilevel structural equation modeling indicator-specific growth model of deep approaches to learning. Retrieved and modified from Geiser et al. (2013).

It was decided, based on later analysis (i.e., ICC ranged from 0.29 to 0.44), that multilevel structure should be considered and MSEM-ISGM was a more appropriate model for analyzing current data. Figure 1 shows the specification of MSEM-IGSM for both levels. It is important to note that data collection intervals were not consistent between waves, with a half year interval from wave 1 to wave 2, a year from wave 2 to wave 3, and 2 years from wave 3 to wave 4, respectively. Due to this variability, the loadings on the slope factors were set to 0, 1, 3, and 7 to define a linear growth model reflected actual time intervals within level 1.

In Figure 1, level 1 represented the measurements at the different time points that are nested within the Level-2 units (individuals). The trait effects of the persons were captured on Level 2. On level 1, t represented time point of the deep approaches to learning, and y1–y4 represented four items for measuring deep approaches to learning. Residual variables (ε_*i*_) that reflected random measurement errors of the four items. The effect of measurement errors captured by the variances of residual variables (σ^2^_ε_). Each item simultaneously shared a common latent trait factor (ξ) and a latent state residual factor (ζ). Latent trait factor reflects the effect of individual differences, whereas latent state residual factor characterizes effects of the situation. The state residual factor loadings (γ_*i*_) are set to allow for potential differences between indicators. The variance of the common latent state residual factor (σ^2^_ζ_) captures the effects of the situation on deep approaches to learning, and it was used to clarify hypothesis 1. The intercepts and slope of the trait factor (ξ_*int*_ and ξ_*slope*_) are, respectively, the initial status and weights for each items at each time point. The black dots between the arrow pointing from the time variable (t) to the indicators mean that they (including their variances) are allowed to vary across individuals. To identify the model, the latent state residual factor loading parameter (γ_1_) of one reference indicator (y1) was set to 1. All factor loadings on the intercept factors must be set to 1 in the model, and all factor loadings on the slope factors should be set to *t*. The remaining state residual factor loadings, variances of all latent variables and means of the latent intercept and slope factors, as well as their co-variances were identified and can be estimated as free parameters.

On level 2, no common factors were introduced, the latent trait means E (ξ) and variances Var (ξ) of the intercepts and slope of the trait factor are estimated. ξ_*int*_ represents the latent trait scores at each time, while ξ_*slope*_ denotes the latent differences between the trait scores at time t and the trait scores at t-1. The means E (ξ_*intercept*_) and variances Var (ξ_*intercept*_) of the intercepts were used to examine hypothesis 2. The means of the intercepts larger than and equal to 3 indicated that the states of the deep approaches to learning was high, whereas the significant variances of the intercepts and their values above 0.20 implied the deep approaches to learning showed considerable individual differences. The means E (ξ_*slope*_) and variances Var (ξ_*slope*_) of the intercepts were used to examine hypothesis 3 (Geiser et al., 2013). The means and variances of the slopes, respectively, reflects the change/trend and stability of the deep approaches to learning from kindergarten to third grade. Analyses were performed in Mplus, and the Full Information Maximum Likelihood (FIML) method was used to appropriately handle missing data (Muthén and Muthén, 2012). This approach was used because it produced parameter estimates that were less biased than list-wise deletion, pairwise deletion or mean imputation (Enders, 2001).

## Results

### Preliminary Analysis

Multivariate descriptive statistics were presented in Table 1. Descriptive analyses were conducted to describe each item of deep approaches to learning at four time points. As shown in Table 1, kindergarteners’ deep approaches to learning were high (ranging from 2.91 to 3.12) in the fall of kindergarten. Students showed a similar mean level (ranging from 2.94 to 3.23) in the following three time points. This seems to suggest that, on average, deep approaches to learning were quite stable from kindergarten through to third grade.

**TABLE 1:** Descriptive statistics for each item of deep approaches to learning from kindergarten to third grade.

Time	The fall of kindergarten		The spring of kindergarten		The spring of first grade		The spring of third grade	
	(wave 1)		(wave 2)		(wave 3)		(wave 4)	
	(*N* = 19,213)		(*N* = 19,271)		(*N* = 15,048)		(*N* = 11,760)	

	M	SD	M	SD	M	SD	M	SD
Item 1	3.12	0.83	3.23	0.82	3.19	0.83	3.09	0.85
Missing (%)	10.58		10.20		29.76		45.08	
Item 2	2.99	0.85	3.15	0.85	3.12	0.86	3.14	0.84
Missing (%)	10.44		10.11		29.70		45.02	
Item 3	2.99	0.87	3.14	0.87	3.06	0.90	3.05	0.90
Missing (%)	10.67		10.22		29.90		45.16	
Item 4	2.91	0.87	3.02	0.87	2.97	0.89	2.94	0.89
Missing (%)	10.40		10.12		29.75		45.13	

Additional analysis was performed to examine whether there were considerable similarities among the four measurements. Results showed that the ICCs for the four items were 0.29, 0.38, 0.36, and 0.44, respectively; all of which are above the criteria of 0.05 suggested by researchers (Heck, 2001; Dyer et al., 2005). These findings imply that a two-level structure should be considered in the following analysis.

### Appropriate Model for Evaluating the States, Stability, and Changes of Deep Approaches to Learning

Before identifying the appropriate model for evaluating the states, stability, and changes of deep approaches to learning, the longitudinal measurement invariance of the four items were examined. Four invariance model were analyzed, they were the configural invariance model, the metric invariance model, the scalar invariance model, and the strict invariance model. Differences of CFI (ΔCFI) and TLI (ΔTFI) between two models, respectively, with standards of ≤0.01 and ≤0.02 were considered invariance between less strict and stricter models (Cheung and Rensvold, 2002). Results showed that the CFI and TLI were, respectively, 0.957 and 0.945 for the configural invariance model, 0.954 and 0.948 for the metric invariance model, 0.944 and 0.943 for the scalar invariance model, and 0.945 and 0.949 for strict invariance model. The all ΔCFIs and ΔTFI between the former (less strict) model and the latter (stricter) model were ≤0.01 and <0.02. It clearly indicated that the longitudinal measurement invariance was hold, suggesting that the results of MSEM-ISGM are guaranteed.

### The Means and Variances of the States, Stability, and Changes in Deep Approaches to Learning

In each of the two levels, different effects were modeled and analyzed. Within level 1, three different effects were modeled and analyzed, the first of which being the time-invariant factor loadings γ_*i*_. The second effect was the common latent state residual factor that is shared between indicators as represented by the time-invariant variance parameter Var (ζ). The last effect was of measurement error as represented by the time-invariant error variance parameters Var (ε_*i*_). Within level 2, the effects of time, as represented by the random intercepts ξint_*i*_ and the random slopes ξslope_*i*_ were analyzed.

#### The Effect of Learning Context on Deep Approaches to Learning

Table 2 presents all the unstandardized parameters estimated in MSEM-ISGM. As shown, the unstandardized state residual factor loadings for item 1 to item 4 was 1.000, 1.103, 1.158, and 1.065, respectively, with a factor variance of 0.206. These results suggest that there was only a part effect of situation on the deep approaches to learning. Instead, variances were mostly found in the trait of deep approaches to learning, which is not easily affected by context. The unstandardized error variances were 0.262, 0.195, 0.210, and 0.183, respectively, clearly showing that errors variances for the four items should be considered. Considering both unstandardized residual factor variances and error variances, which imply approximately 20% and 80% of variations in items for the deep approaches to learning, these can be, respectively, explained by the contextual factors and the trait. These findings demonstrate the considerable (rather than trivial) effect of the contextual factors on the deep approaches to learning. Hence, the hypothesis 1 was not supported by this finding.

**TABLE 2:** Parameter estimates and standard errors for the multilevel structural equation modeling indicator-specific growth model.

Parameter label	Parameter	Estimates	*SE*
**Level 1**			
State residual factor loadings	γ_1_	1.000*	0.000
	γ_2_	1.103*	0.017
	γ_3_	1.158*	0.019
	γ_4_	1.065*	0.017
State residual factor variances	var (ζ)	0.206*	0.006
Error variances	var (ε_1_)	0.262*	0.008
	var (ε_2_)	0.195*	0.007
	var (ε_3_)	0.210*	0.008
	var (ε_4_)	0.183*	0.007
**Level 2**			
Factor means	*E* (ξ_*intercept1*_)	3.181*	0.006
	*E* (ξ_*intercept2*_)	3.055*	0.007
	*E* (ξ_*intercept3*_)	3.062*	0.007
	*E* (ξ_*intercept4*_)	2.970*	0.007
	*E* (ξ_*slope1*_)	-0.015*	0.002
	*E* (ξ_*slope2*_)	0.015*	0.002
	*E* (ξ_*slope3*_)	-0.007*	0.002
	*E* (ξ_*slope4*_)	-0.009*	0.002
Factor variances	var (ξ_*intercept1*_)	0.221*	0.001
	var (ξ_*intercept1*_)	0.287*	0.001
	var (ξ_*intercept1*_)	0.271*	0.001
	var (ξ_*intercept1*_)	0.352*	0.001
	var (ξ_*slope1*_)	0.004*	0.012
	var (ξ_*slope2*_)	0.001	0.011
	var (ξ_*slope3*_)	0.002	0.013
	var (ξ_*slope4*_)	0.002*	0.011

* p < 0.05.

#### The State, Stability, and Change of the Deep Approaches to Learning

On an individual level (level 2), the overall means of intercept in each item of approaches to learning were 3.181, 3.055, 3.062, and 2.970, respectively. The overall variances of intercept in each item were 0.221, 0.287, 0.271, and 0.352, respectively. It indicated that the states of the deep approaches to learning showed considerable variations, it reflected considerable individual differences among children from kindergarten to third grade. The hypothesis 2 was supported because the deep approach to learning was relatively high from kindergarten to third grade.

The overall means of slope for items were −0.015, 0.015, −0.007, and −0.009, respectively. Most of them (except for item 2) showed a small but significant decline over time, whereas item 2 revealed the only overall positive growth. The overall variance of slope for items were 0.004, 0.001, 0.002, and 0.002, respectively. The half of items exhibiting small variation (except item 2 and item 3). Altogether, it implied that the general trend of deep approaches to learning was relatively constant. It suggested the deep approaches to learning was relatively stable. As a result, the hypothesis 3 was supported.

## Discussion

The main purpose of this study was to investigate the effect of context on deep approaches to learning as well as the states and changes of which for children from kindergarten to third grade. The similarities among measurements were examined for the rationality of considering a multilevel structure. It would be problematic if the traditional latent growth model was applied to analyze deep approaches to learning. This was because of the considerable measurement errors within the four items, which suggests a single composite score is not statistically valid. Therefore. The MSEM-ISGM was used as a more appropriate model to examine the proposed hypotheses of this study.

It may be the case that students are inherently or intrinsically motivated to engage in, pay attention to, and work persistently on learning a task until completion. However, the considerable state residual factor variances suggest that deep approaches to learning may be partly affected by learning context (i.e., class activity) (Goto et al., 2018). Therefore, it is also possible that the eagerness to learn, work independence, task persistence, and attentiveness may partly reflect situational interest (Krapp et al., 1992; Hidi and Renninger, 2006). Situational interest refers to a student’s focused attention and affective reaction to a task that is triggered temporarily by environmental stimuli (such as the presence of novel work), which may or may not last over time (Hidi, 1990). It is apparent that kindergarteners or pupils are attracted to novel tasks due to the induced enjoyment, which can then induce a willingness to learn, increased attentiveness and a desire to complete the task independently.

Regarding the states, stability and changes of approaches to learning, the construct remained relatively high and relative stable from kindergarten to third grade, which was consistent with hypothesis 2 and 3. These findings were also consistent with results in the field of learning motivation (e.g., intrinsic motivation and goal motivation). For example, development research has indicated that children’s mastery goal orientation remains high (Rholes et al., 1980; Dweck and Elliot, 1983; Harter, 2012; Laitinen et al., 2017), even after failure (Rholes et al., 1980; Dweck and Elliot, 1983; Harter, 2012). This motivational focus may prompt active engagement in learning a task and the adoption of adaptive motivational strategies, such as persistence (Schunk et al., 2008). However, this study also identified considerable variations in the intercepts of the deep approaches to learning. This implies that individual differences in deep approaches to learning was considerable and consistent across the four time points. Interestingly, it was found that the overall means of slope were consistent in the same negative direction, except for the item regarding independent work. These results were a little inconsistent to Sung and Wickrama (2018)’s finding. These results suggest that eagerness to learn, persistence and attention declined from kindergarten to third grade. It may also infer that intrinsic motivation, interest, and mastery goal orientation begins to decrease from kindergarten, which is earlier than expected by researchers’ within the current motivational literature (Meece and Miller, 2001; Lepper et al., 2005; Bong, 2009; Tuominen-Soini et al., 2011). Contrasting to this, teachers observed that young children increasingly preferred to work independently. This appears to be consistent with the practical observation that most kindergarteners tend to complete learning tasks collaboratively. Researchers have also found that students’ need for autonomy increases with age (Jang et al., 2012). Students may therefore fulfill this increased need for autonomy through the independent completion of learning tasks (Schunk et al., 2008). As a result, the growth of independent work observed in this study may be explained by the increased need of autonomy proposed in the self-determination theory (Ryan and Deci, 2000).

Finally, considering data-gathering process in this study, the approaches to learning for each children was evaluated by their teacher according to teacher’s daily observations and general impressions to that children. It was because self-report by young children using typical questionnaire was considered to be challenging due to the tendency to be highly positive in ratings of their own inner activities (i.e., abilities and interests) and the restriction on text reading and comprehension abilities (George and Greenfield, 2005). However, young children themselves should also be considered as important sources of information because they may be able to report themselves by using measurement they are familiar with and are understandable (Wu, 2022). This was especially important when internal constructs of the many aspects of approaches to learning are including (i.e., persistence, and preference for challenge) (George and Greenfield, 2005). The appropriate instrument for young children to self-report their approaches to learning was absent. Measurement for approaches to learning with familiar and understandable content for young children is clear needed. It was beneficial for giving researchers better understanding about approaches to learning for young children from different sources of information.

## Implications

The results of this study may have some theoretical and practical implications, particularly in regard to the relationship between approaches to learning and motivation. Approaches to learning may be an explicit representation of motivational belief, just as achievement goal theory originates from sense of competence (Elliot, 2005). Within achievement goal theory, a mastery goal orientation can create the desire to learn, with students being more able to persistently concentrate on a task. Consistent with findings in the motivational literature, the decline of this type of motivation may reflect the decrease in most deep approaches to learning. Specifically, the decline in student’s mastery goal orientation may signal the diminishing adaptation of the deep approaches to learn, imbedded in a sense of competence. However, this seems contrary to the prediction of achievement goal theory due to the increase in the desire to work independently. This may be explained by the self-determination theory (SDT), which argues that students need for autonomy generally increases with age. Thus, the development of increased autonomy may be independent to that of mastery goal orientation which is rooted in the need for competence. The findings of this study may therefore provide supportive evidence that most deep approaches to learning is rooted in the need for competence, which is different to the origins of the need for autonomy, perhaps developing parallel to each other.

In the past, related research adopted the use of a composite score to represent a student’s approach to learning. However, this study identified variances in each item and states score traditionally used to form the single score. In addition, items were not changing in the same direction over time. Therefore, variances from different sources were confounded in conventional growth modeling, preventing the examination of situational effects. As a result, it was determined that items should be analyzed separately to decompose the variation contributed by measurement error, context and trait. Hence, indicator-specific growth modeling was adopted for this kind of analysis.

Another limitation identified in past research includes the structure in which time nested in individuals. Within conventional approaches, there was the assumption that the states, stability, and changes in deep approaches to learning contained no variation. However, it was evident within the current study that there were statistically significant variations around the means of most intercepts and slopes. The nested structure should therefore be considered through a multilevel SEM approach. Altogether, compared to conventional LGM, the MSEM-ISGM was superior in decomposing different effects and therefore investigating the states, stability and changes in approaches to learning. Due to the appropriateness of MSEM-ISEM, past research concerning longitudinal relationships involving deep approaches to learning may need to be re-examined.

For practitioners, the overall decline in deep approaches to learning combined with the constant variabilities around the means of the four items may imply two conditions. Firstly, it can be inferred that education in both kindergarten and early primary school exerts a positive effect on children, but this effect is continually attenuated by the detrimental effect of time-varying factors such as instruction. Secondly, the effect of context hold constant while learning tasks or material becomes boring to the student, therefore the task content becomes insufficient in eliciting intrinsic motivation, interest, or mastery motivation (Schunk et al., 2008). In general, family factors are relatively stable, while the learning context in school is changing for children who transited from kindergarten to primary school and from early to middle elementary school. Specifically, as children carry-out elementary school, task difficulty increases as well. As students have increased opportunities to master learning content independently, it is reasonable to infer that they may experience an increased number of failures in the learning process, which attenuates their eagerness to learn and decreases persistence and attentiveness to the task (Dickhauser et al., 2011).

In practice, teachers should begin to monitor students’ learning process from early childhood, because their adaptive motivation and learning strategy may start to decline from kindergarten. Due to a need for relatedness, kindergarten children tend to prefer working cooperatively (Deci and Ryan, 2000) which could be used to trigger students’ situational interest or social goal (Urdan and Maehr, 1995). Thus, cooperative learning may in turn exert a positive effect on adaptive motivation and strategy. This suggests that teachers working within the early stages of elementary school should consider more cooperative learning activities to promote adaptive motivation and strategy.

## Summary

This study aimed to realize the utility of considering multilevel multiple indicator growth modeling, and to examine the effect of the context as well as the states, stability, and changes in deep approaches to learning. Results documented the advantage of MSEM-ISGM over conventional growth modeling. The effect of context on deep approaches to learning was also supported. In addition, the states and changes of the deep approaches to learning from kindergarten to third grade was also demonstrated, which partly supported and challenged former researches. For instance, the findings of this study complement former findings within the motivational literature, supporting the possible relationship between deep approaches to learning and a mastery goal orientation. On this basis, future research may need to investigate the relationship between other variables and deep approaches to learning. For practitioners, the results of this study may increase awareness of the possible decline in adaptive motivation and learning strategies, encouraging the adoption of appropriate interventions for prevention.

## Limitations of the Study

The current study focused on the use of a more appropriate method for decomposing the effect from measurement errors and investigating the states, stability, and changes in deep approaches to learning. Due to this focus, there were no other variables included in the analysis. It is evident that future research needs to re-examine the effects of deep approaches to learning on other variables (e.g., achievement scores) by using either single or multilevel SEM-based ISGM. Moreover, the lack of variables regarding learning motivation (e.g., intrinsic motivation, interest, or achievement goal) resulted in the inability to investigate the effect of motivational variables on deep approaches to learning. This may restrict particular inferences of this study. Another limitation of this study was that kindergarteners and young pupils were unable to rate themselves on the items of deep approaches to learning. As a result, the assessment relied on the observations of their teachers. For future research, it is necessary to develop an instrument which grants young children the ability to report by themselves or participate in experimental activities. It may also be beneficial to compare rating consistency between teachers and children.

In addition, the analysis approach of this study was variable-based rather than person-based. The person-based approach could have been used to investigate the pattern or profile of deep approaches to learning at each time point as well as pattern changes over time. Specifically, kindergarteners with similar profiles of deep approaches to learning could be clustered at each of four time points from kindergarten to third grade, with different subgroups containing their own profiles. Latent transition could be used to investigate the changes of these profiles over time. Further, other variables could be included to investigate their effect on the profile changes. Since this approach was out of the scope of this study, future research should address this issue using a person-based analysis approach. It should be noted that the single level person-based approach became increasingly computationally demanding as subjects and model complexity increased.

## Data Availability Statement

The original contributions presented in the study are included in the article, further inquiries can be directed to the corresponding author.

## Author Contributions

The author confirms being the sole contributor of this work and has approved it for publication.

## Conflict of Interest

The author declares that the research was conducted in the absence of any commercial or financial relationships that could be construed as a potential conflict of interest.

## Publisher’s Note

All claims expressed in this article are solely those of the authors and do not necessarily represent those of their affiliated organizations, or those of the publisher, the editors and the reviewers. Any product that may be evaluated in this article, or claim that may be made by its manufacturer, is not guaranteed or endorsed by the publisher.

## References

[B1] Anderman E. M. Midgley C. (1997). Changes in achievement goal orientations, perceived academic competence, and grades across the transition to middle-level schools. *Contemp. Educ. Psychol.* 22 269–298. 10.1006/ceps.1996.0926 9237829

[B2] Ardura D. Galán A. (2019). The interplay of learning approaches and self-efficacy in secondary school students’ academic achievement in science. *Int. J. Sci. Educ.* 41 1723–1743.

[B3] Baeten M. Kyndt E. Struyven K. Dochy F. (2010). Using student-centred learning environments to stimulate deep approaches to learning: Factors encouraging or discouraging their effectiveness. *Educ. Res. Rev.* 5 243–260. 10.1016/J.EDUREV.2010.06.001

[B4] Betts L. R. Rotenberg K. J. (2007). A short form of the teacher rating scale of school adjustment. *J. Psychoeduc. Assess.* 25 150–164. 10.1177/0734282906296406

[B5] Biggs J. (1988). The role of metacognition in enhancing learning. *Aus. J. Educ.* 32 127–138. 10.1177/000494418803200201

[B6] Biggs J. Kember D. Leung D. Y. P. (2001). The revised two-factor study process questionnaire: R-SPQ-2F. *Br. J. Educ. Psychol.* 71 133–149. 10.1348/000709901158433 11307705

[B7] Biggs J. B. Moore P. J. (1993). *The Process of Learning*, 3rd Edn. Hoboken, NJ: Prentice Hal

[B8] Blair C. (2002). School readiness as propensity for engagement: Integrating cognition and emotion in a neurobiological conceptualization of child functioning at school entry. *Am. Psychol.* 57 111–127. 10.1037/0003-066X.57.2.111 11899554

[B9] Bong M. (2009). Age-related differences in achievement goal differentiation. *J. Educ. Psychol.* 101 879–896. 10.1037/A0015945

[B10] Bouffard T. Marcoux M. F. Vezeau C. Bordeleau L. (2003). Changes in self-perceptions of competence and intrinsic motivation among elementary school children. *Br. J. Educ. Psychol.* 73 171–186. 10.1348/00070990360626921 12828811

[B11] Chan A. K. M. Botelho M. G. Lam O. L. T. (2021). The relation of online learning analytics, approaches to learning and academic achievement in a clinical skills course. *Euro. J. Dent. Educ.* 25 442–450. 10.1111/eje.12619 33185309

[B12] Cheung G. W. Rensvold R. B. (2002). Evaluating goodness-of-fit indexes for testing measurement invariance. *Struct. Equ. Model*. 9, 233–255. 10.1207/s15328007sem0902_5 33486653

[B13] Deci E. L. Ryan R. M. (2000). The “what” and “why” of goal pursuits: Human needs and the self-determination of behavior. *Psychol. Inquiry* 11 227–268. 10.1207/s15327965pli1104_01

[B14] Dickhauser C. Buch S. R. Dickhauser O. (2011). Achievement after failure: The role of achievement goals and negative self-related thoughts. *Learn. Instruct.* 21 152–162. 10.1016/j.learninstruc.2010.01.002

[B15] Duff A. Boyle E. Dunleavy K. Ferguson J. (2004). The relationship between personality, approach to learning and academic performance. *Pers. Individ. Dif.* 36 1907–1920. 10.1016/J.PAID.2003.08.020

[B16] Dweck C. S. Elliot E. S. (1983). “Achievement motivation,” in *Handbook of Child Psychology: Social and Personality Development*, Vol. Vol. 4 ed. Hetherington E. M. (New York: Wiley).

[B17] Dyer N. G. Hanges P. J. Hall R. J. (2005). Applying multilevel confirmatory factor analysis techniques to the study of leadership. *Lead. Quar.* 16 149–167. 10.1016/J.LEAQUA.2004.09.009

[B18] Elliot A. J. (2005). “A conceptual history of the achievement goal construct,” in *Handbook of Competence and Motivation*, eds Elliot A. J. Dweck C. S. (New York: Guilford Press), 55–72.

[B19] Enders C. K. (2001). The performance of the full information maximum likelihood estimator in multiple regression models with missing data. *Educ. Psychol. Meas.* 61 713–740. 10.1177/0013164401615001

[B20] Entwistle N. J. McCune V. Walker P. (2000). “Conceptions, styles and approaches within higher education: Analytic abstractions and everyday experience,” in *Perspectives on Cognitive, Learning, and Thinking Styles*, eds Sternberg R. J. Zhang L. F. (Mahwah NJ: Lawrence Erlbaum), 10.4324/9781410605986

[B21] Fryer L. K. Vermunt J. D. (2018). Regulating approaches to learning: Testing learning strategy convergences across a year at university. *Br. J. Educ. Psychol.* 88 21–41. 10.1111/bjep.12169 28691734

[B22] García T. Rodríguez C. Betts L. Areces D. González-Castro P. (2016). How affective-motivational variables and approaches to learning predict mathematics achievement in upper elementary levels. *Learn. Indiv. Dif.* 49 25–31. 10.1016/j.lindif.2016.05.021

[B23] Geiser C. Bishop J. Lockhart G. Shiffman S. Grenard J. L. (2013). Analyzing latent state-trait and multiple-indicator latent growth curve models as multilevel structural equation models. *Front. Psychol.* 4:975. 10.3389/fpsyg.2013.00975 24416023 PMC3874722

[B24] George J. L. Greenfield D. B. (2005). Examination of a structured problem-solving flexibility task for assessing approaches to learning in young children: Relation to teacher ratings and children’s achievement. *Appl. Devel. Psychol.* 26 69–84. 10.1016/j.appdev.2004.10.006

[B25] Goh P. S. C. Wong K. T. Osman R. (2012). Student-teachers’ approaches to learning, academic performance and teaching efficacy. *Malaysian J. Learn. Instruct*. 9, 31–46. 10.32890/mjli.9.2012.7635

[B26] Goto T. Nakanishi K. Kano K. (2018). A large-scale longitudinal survey of participation in scientific events with a focus on students’ learning motivation for science: Antecedents and consequences. *Learn. Indiv. Dif.* 61 181–187. 10.1016/j.lindif.2017.12.005

[B27] Gottfried A. E. Fleming J. S. Gottfried A. W. (2001). Continuity of academic intrinsic motivation from childhood through late adolescence: A longitudinal study. *J. Educ. Psychol.* 93 3–13. 10.1037/0022-0663.93.1.3

[B28] Greer F. W. DiStefano C. A. Liu J. Cain L. K. (2015). Preliminary psychometric evidence of the behavioral and emotional screening system teacher rating scale–Preschool. *Psychom. Rep.* 40 240–246. 10.1177/1534508415571594

[B29] Gresham P. M. Elliot S. N. (1990). *Social Skills Rating System.* Minnesota: American Guidance Service, 10.1037/T10269-000

[B30] Harter S. (1981). A new self-report scale of intrinsic versus extrinsic orientation in the classroom: Motivational and informational components. *Devel. Psychol.* 17 300–312. 10.1037/0012-1649.17.3.300

[B31] Harter S. (2012). *the Construction of the Self: Developmental and Sociocultural Foundations*, 2nd Edn. New York: Guilford Press, 10.5860/choice.50-1160

[B32] Heck H. R. (2001). “Multilevel Modeling with SEM,” in *New Developments and Techniques in Structural Equation Modeling*, eds Marcoulides G. A. Schumacker R. E. (Mahwah: Lawrence Erlbaum Associates), 89–127. 10.4324/9781410601858

[B33] Hidi S. (1990). Interest and its contribution as a mental resource for learning. *Rev. Educ. Res.* 60 549–591. 10.3102/00346543060004549

[B34] Hidi S. Renninger A. (2006). The four-phase model of interest development. *Educ. Psychol.* 41 111–127. 10.1207/s15326985ep4102_4

[B35] Hu X. Yeo G. B. (2020). Emotional exhaustion and reduced self-efficacy: The mediating role of deep and surface learning strategies. *Motiv. Emot.* 44 785–795.

[B36] Jang H. Kim E. J. Reeve J. (2012). Longitudinal test of self-determination theory’s motivation mediation model in a naturally occurring classroom context. *J. Educ. Psychol.* 104 1175–1188. 10.1037/A0028089

[B37] Kaplan D. (2009). *Structural Equation Modeling: Foundations and Extensions*, 2nd Edn. New York: Sage, 10.4135/9781452226576

[B38] Khan A. H. Kulkarni S. Mahmood T. Khan A. (2018). Evidence-based approaches to learning. *Adv. Med. Educ. Pract.* 16 581–582. 10.2147/AMEP.S171499 30147390 PMC6101737

[B39] Krapp A. Hidi S. Renninger K. A. (1992). “Interest, learning and development,” in *The Role of Interest in Learning and Development*, eds Renninger K. A. Hidi S. Krapp A. (Mahwah, NJ: Lawrence Erlbaum Associates), 3–25. 10.4324/9781315807430

[B40] Laitinen S. Lepola J. Vauras M. (2017). Early motivational orientation profiles and language comprehension skills: From preschool to Grade 3. *Learn. Indiv. Dif.* 53 69–78. 10.1016/J.LINDIF.2016.11.002

[B41] Lepper M. R. Corpus J. H. Iyengar S. S. (2005). Intrinsic and extrinsic motivational orientations in the classroom: Age differences and academic correlates. *J. Educ. Psychol.* 97 184–196. 10.1037/0022-0663.97.2.184

[B42] Li-Grining C. P. Votruba-Drzal E. Maldonado-Carreño C. Haas K. (2010). Children’s early approaches to learning and academic trajectories through fifth grade. *Devel. Psychol.* 46 1062–1077. 10.1037/a0020066 20822223

[B43] McClelland M. M. Acock A. C. Morrison F. J. (2006). The impact of kindergarten learning-related skills on academic trajectories at the end of elementary school. *Early Childhood Res. Quart.* 21 471–490. 10.1016/J.ECRESQ.2006.09.003

[B44] McDermott P. A. Leigh N. M. Perry M. A. (2002). Development and validation of the preschool learning behaviors scale. *Psychol. Schools* 39 353–365.

[B45] McWatt S. C. Newton G. S. Umphrey G. J. Jadeski L. C. (2021). Dissection versus prosection: A comparative assessment of the course experiences, approaches to learning, and academic performance of non-medical undergraduate students in human anatomy. *Anatom. Sci. Educ.* 14 184–200. 10.1002/ase.1993 32539226

[B46] McWayne C. M. Fantuzzo J. W. McDermott P. A. (2004). Preschool competency in context: An investigation of the unique contribution of child competencies to early academic success. *Devel. Psychol.* 40 633–645. 10.1037/0012-1649.40.4.633 15238049

[B47] Meece J. L. Miller S. D. (2001). A longitudinal analysis of elementary school students’ achievement goals in literacy activities. *Contemp. Educ. Psychol.* 26 454–480. 10.1006/CEPS.2000.1071 11681828

[B48] Morgan P. L. Farkas G. Wu Q. (2011). Kindergarten children’s growth trajectories in reading and mathematics: Who falls increasingly behind? *J. Learn. Disabil.* 44 472–488. 10.1177/0022219411414010 21856991 PMC4554713

[B49] Muthén L. K. Muthén B. O. (2012). *Mplus User’s Guide*, 7th Edn. Los Angeles: Muthén & Muthén.

[B50] National Center for Education Statistics (2001). *Early childhood longitudinal study, kindergarten class of 1998–99: Base year public-use data files user’s manual (NCES 2001–029).* Washington, D.C: National Center for Education Statistics.

[B51] Piumatti G. Abbiati M. Gerbase M. W. Baroffio A. (2021). Patterns of change in approaches to learning and their impact on academic performance among medical students: Longitudinal analysis. *Teach. Learn. Med.* 33 173–183. 10.1080/10401334.2020.1814295 33023316

[B52] Rathbun A. West J. (2004). *From Kindergarten Through Third Grade: Children’s Beginning School Experiences.* Washington, D.C: Center for Education Statistics.

[B53] Razza R. A. Martin A. Brooks-Gunn J. (2015). Are approaches to learning in Kindergarten associated with academic and social competence similarly? *Child Youth Care Forum* 44 757–776. 10.1007/s10566-015-9307-0 26877624 PMC4749025

[B54] Rentzios C. Kamtsios S. Karagiannopoulou E. (2019). The mediating role of implicit and explicit emotion regulation in the relationship between academic emotions and approaches to learning: Do defense styles matter? *J. Nervous and Mental Dis.* 207 683–692. 10.1097/NMD.0000000000001027 31356408

[B55] Rholes W. S. Blackwell J. Jordan C. Walters C. (1980). A developmental study of learned helplessness. *Devel. Psychol.* 16 616–624. 10.1037/0012-1649.16.6.616

[B56] Ryan R. M. Deci E. L. (2000). Intrinsic and extrinsic motivations: Classic definitions and new directions. *Contemp. Educ. Psychol.* 25 54–67. 10.1006/ceps.1999.1020 10620381

[B57] Schunk D. H. Pintrich P. R. Meece J. L. (2008). *Motivation in education: Theory, Research, and Applications.* Pearson: Merrill Prentice Hall.

[B58] Sideridis G. D. (2005). Goal orientation, academic achievement, and depression: Evidence in favor of a revised goal theory framework. *J. Educ. Psychol.* 97 366–375. 10.1037/0022-0663.97.3.366

[B59] Sung J. Wickrama K. A. S. (2018). Longitudinal relationship between early academic achievement and executive function: Mediating role of approaches to learning. *Contemp. Educ. Psychol.* 54 171–183. 10.1016/j.cedpsych.2018.06.010

[B60] Teoh H. C. Abdullah M. C. Roslan S. Daud S. M. (2014). Assessing students approaches to learning using a matrix framework in a Malaysian public university. *Springer Plus* 3 54–64. 10.1186/2193-1801-3-54 24600539 PMC3935033

[B61] Tourangeau K. Nord C. Lê T. Sorongon A. G. Najarian M. (2009). *Early childhood longitudinal study, kindergarten class of 1998–99 (ECLS-K), combined user’s manual for the ECLS-K eighth-grade and K-8 full sample data files and electronic codebooks (NCES 2009-004)* Washington DC: National Center for Education Statistics

[B62] Tuominen-Soini H. Salmela-Aro K. Niemivirta M. (2011). Stability and change in achievement goal orientations: A person-centered approach. *Contemp. Educ. Psychol.* 36 82–100. 10.1016/J.CEDPSYCH.2010.08.002

[B63] Urdan T. C. Maehr M. L. (1995). Beyond a two-goal theory of motivation and achievement: A case for social goals. *Rev. Educ. Res.* 65 213–243. 10.3102/00346543065003213

[B64] von Oerzen T. Hertzog C. Lindenberger U. Ghisletta P. (2010). The effect of multiple indicators on the power to detect inter-individual differences in change. *Br. J. Math. Stat. Psychol.* 63 627–646. 10.1348/000711010X486633 20211053

[B65] Wang J. Wang X. (2012). *Structural equation modeling: Application using Mplus.* Hoboken: Wiley.

[B66] Wu C. C. (2022). Examining the effectiveness and efficiency of an innovative achievement goal measurement for preschoolers. *Front. Psychol.* 12:741088. 10.3389/fpsyg.2021.741088 35069320 PMC8766295

[B67] Zakariya Y. F. Nilsen H. K. Bjørkestøl K. Goodchild S. (2021). Analysis of relationships between prior knowledge, approaches to learning, and mathematics performance among engineering students. *Int. J. Math. Educ. Sci. Technol*, 1–19 10.1080/0020739X.2021.1984596

[B68] Zakariya Y. F. Nilsen H. K. Goodchild S. Bjørkestøl K. (2020). Self-efficacy and approaches to learning mathematics among engineering students: Empirical evidence for potential causal relations. *Int. J. Math. Educ. Sci. Technol.* 2020 1–15 10.1080/0020739X.2020.1783006

[B69] Zeegers P. (2001). Approach to learn in science: A longitudinal study. *Br. J. Educ. Psychol.* 71 115–132. 10.1348/000709901158424 11307704

